# Gait variability as digital biomarker of disease severity in Huntington’s disease

**DOI:** 10.1007/s00415-020-09725-3

**Published:** 2020-02-11

**Authors:** Heiko Gaßner, Dennis Jensen, F. Marxreiter, Anja Kletsch, Stefan Bohlen, Robin Schubert, Lisa M. Muratori, Bjoern Eskofier, Jochen Klucken, Jürgen Winkler, Ralf Reilmann, Zacharias Kohl

**Affiliations:** 1 Department of Molecular Neurology, University Hospital Erlangen, Friedrich-Alexander University Erlangen-Nürnberg (FAU), Schwabachanlage 6, 91054 Erlangen, Germany; 2 George-Huntington Institute (GHI) GmbH, Münster, Germany; 3 Rehabilitation Research and Movement Performance Laboratory (RRAMP Lab), Stony Brook University, Stony Brook, NY USA; 4 Machine Learning and Data Analytics Lab, Friedrich-Alexander University Erlangen-Nürnberg (FAU), Erlangen, Germany; 5 Medical Valley-Digital Health Application Center GmbH, Bamberg, Germany; 6 Fraunhofer Institute for Integrated Circuits IIS, Erlangen, Germany; 7 Department of Radiology, University of Muenster, Muenster, Germany; 8 Department of Neurodegenerative Diseases and Hertie-Institute for Clinical Brain Research, University of Tuebingen, Tuebingen, Germany; 9 Center for Rare Diseases Erlangen, University Hospital Erlangen, Erlangen, Germany; 10 Department of Neurology, University of Regensburg, Regensburg, Germany

**Keywords:** Huntington’s disease, Gait analysis, Wearable sensors, Gait variability, Regularity of gait

## Abstract

**Background:**

Impaired gait plays an important role for quality of life in patients with Huntington’s disease (HD). Measuring objective gait parameters in HD might provide an unbiased assessment of motor deficits in order to determine potential beneficial effects of future treatments.

**Objective:**

To objectively identify characteristic features of gait in HD patients using sensor-based gait analysis. Particularly, gait parameters were correlated to the Unified Huntington’s Disease Rating Scale, total motor score (TMS), and total functional capacity (TFC).

**Methods:**

Patients with manifest HD at two German sites (*n* = 43) were included and clinically assessed during their annual ENROLL-HD visit. In addition, patients with HD and a cohort of age- and gender-matched controls performed a defined gait test (4 × 10 m walk). Gait patterns were recorded by inertial sensors attached to both shoes. Machine learning algorithms were applied to calculate spatio-temporal gait parameters and gait variability expressed as coefficient of variance (CV).

**Results:**

Stride length (− 15%) and gait velocity (− 19%) were reduced, while stride (+ 7%) and stance time (+ 2%) were increased in patients with HD. However, parameters reflecting gait variability were substantially altered in HD patients (+ 17% stride length CV up to + 41% stride time CV with largest effect size) and showed strong correlations to TMS and TFC (0.416 ≤ *r*_Sp_ ≤ 0.690). Objective gait variability parameters correlated with disease stage based upon TFC.

**Conclusions:**

Sensor-based gait variability parameters were identified as clinically most relevant digital biomarker for gait impairment in HD. Altered gait variability represents characteristic irregularity of gait in HD and reflects disease severity.

**Electronic supplementary material:**

The online version of this article (10.1007/s00415-020-09725-3) contains supplementary material, which is available to authorized users.

## Introduction

Huntington’s disease (HD) is an autosomal-dominant, neurodegenerative disease characterized by the triad of motor deficits, cognitive decline, and neurobehavioral symptoms [1]. In particular, gait impairment plays an important role upon motor functioning as it affects the quality of life, limits the independence of patients with HD, and reduces activities of daily living [2]. From a biomechanical viewpoint, gait is a well-defined movement in humans including regular, cyclic-repetitive sequences [3] making it an ideal kinetic process to be analyzed by instrumented measures. In one of the initial detailed descriptions of HD, Osler noted a “curious irregular gait” [4] without specifying the HD gait irregularity in detail. Measuring gait parameters in patients with HD supports a quantitative and unbiased assessment of motor deficits and provides objective measures to quantify potential beneficial effects of future treatments. Objective gait parameters may provide metric, granular information complementing the Unified Huntington’s disease Rating Scale total motor score (TMS), and total functional capacity (TFC) as established instruments in daily clinical routine evaluating motor impairment and functionality in HD [5].

Irregularity of gait in HD may be evaluated by using objective measures from instrumented gait analysis systems that are able to calculate stride-by-stride variance, in contrast to, e.g. stopwatch measures focusing on mean values. It has been shown that patients with HD walk slower and with smaller steps compared to healthy controls [6–8]. Recent studies using instrumented carpets or 3d motion capture reported results indicating correlations between UHDRS TMS and stride length in a small cohort (*n* = 7) [9] and significant angular changes of the gait cycle (*n* = 30) [10]. Gait variability as a measure of regularity of gait and dynamic postural control seems to be increased in HD [8, 9, 11, 12]. However, these monocentric studies should be interpreted with caution due to small cohorts examined.

Wearable sensors (accelerometers and gyroscopes) combined with machine learning algorithms have shown to provide objective, granular measures that support the rather rater- and time-dependent clinical ratings in neurologic diseases such as Parkinson’s disease [13–16]. Moreover, inertial sensors have the potential to be used in long-term monitoring scenarios at the patients’ home under real-life conditions with the advantage to record gait patterns over several hours instead of very short-lasting periods during an outpatient visit [17–19]. In HD, it has been shown that accelerometer-based sensors differentiate between pre-manifest and manifest HD patients in a cohort of 14 subjects [6]. In particular, sensor-derived velocity, step and stride length were reduced in manifest HD patients. Moreover, machine learning algorithms provide a framework for gait classification to distinguish HD patients from healthy controls [20]. These mobile sensor technologies combined with intelligent algorithms may support the diagnostic workup. However, the clinical relevance of objective parameters provided by wearable systems in comparison to data gained in well-established clinical scores as UHDRS TMS and TFC of HD patients has not been evaluated so far. Furthermore, gait characteristics in HD, particularly irregularity of gait, need to be understood in more depth by clinical validation. The identification of objective sensor-based gait parameters most characteristic for HD gait and reflecting disease severity is still pending.

To address these questions, the aim of the present two-center approach was to objectively assess characteristic features of gait in 43 HD patients compared to age- and gender-matched controls using mobile sensor-based gait analysis. In particular, these gait parameters were correlated to the clinical scores TMS and TFC in order to understand whether objective measures reflect disease severity assessed by clinical rating scales.

## Subjects and methods

Fifty patients with manifest HD were enrolled at two German sites, the Department of Molecular Neurology at the University Hospital Erlangen and the George-Huntington Institute (GHI) GmbH, Münster. HD patients received standardized clinical assessments during their annual Enroll-HD visit including UHDRS-TMS and TFC. Enroll-HD is a worldwide observational study monitoring symptoms and disease progression over time in manifested HD patients or patients at-risk (www.enroll-hd.org). In addition, patients with HD and a cohort of age- and gender matched healthy controls performed a standardized 4 × 10 m walk test [21]. In order to investigate HD patients with manifest motor symptoms solely (TMS > 5), HD patients with TMS ≤ 5 (absence of motor symptoms) were excluded (*n* = 5), two datasets failed due to technical reasons. Thus, 43 datasets were analyzed and compared to 43 controls (Table 1). In 40 HD patients, genetic testing revealed increased Cytosin, Adenin and Guanin (CAG)-trinucleotide expansion. In the remaining patients (*n* = 3), clinically manifest HD symptoms and positive family history were present; however, genetic testing was not performed.

**Table 1:** Characteristics of patients with HD and controls (mean ± SD)

	HD	Controls	*p*
*n*	43	43	
Age (years)	50.0 ± 11.1	51.0 ± 11.3	0.653
Gender (m:f)	25:18	21:22	0.387^†^
Weight (kg)	78.7 ± 20.0	76.9 ± 16.4	0.658
Height (cm)	173.9 ± 9.0	173.7 ± 9.5	0.935
CAG repeats	44.1 ± 4.2 (*n* = 40)	–	–
UHDRS TMS	38.2 ± 17.9	–	–
TFC score	9.1 ± 3.4	–	–
MMSE	27.1 ± 3.2 (*n* = 40)	–	–

Mann–Whitney *U* test. Significance level *p* < 0.05

^†^Chi square test

*CAG repeats* cytosine–adenine–guanine repeats, *UHDRS TMS* Unified Huntington’s Disease Rating Scale total motor score, *TFC* total functional capacity, *MMSE* mini-mental state examination

Gait characteristics were evaluated in a standardized gait test using an instrumented, sensor-based gait analysis system. This system consists of wearable SHIMMER sensors (Shimmer Research Ltd., Dublin, Ireland) laterally attached to the posterior portion of both shoes (Supplementary Figure S1) [22]. Gait signals were recorded within a (tri-axial) accelerometer range of ± 6 g (sensitivity 300 mV/g), a gyroscope range of ± 500°/sec (sensitivity 2 mV/degree/sec), and a sampling rate of 102.4 Hz. Sensor signals were transmitted to a tablet computer via Bluetooth^®^ and stored for subsequent data analysis [13, 23]. Machine learning algorithms were applied to calculate spatio-temporal gait parameters as mean per stride values derived from 4 × 10 m gait tests (e.g. stride length, gait velocity) [21, 24]. Gait variability as a measure of stride-by-stride variance is presented as the coefficient of variance (CV) of each parameter using an average of 40 strides per patient. Participants performed a standardized 4 × 10 m overground gait test on a 10 m-long corridor at both study sites in self-selected walking speed and without stops at turning points. Only straight strides were automatically detected by the stride detection algorithm [21] and used for gait parameter calculations as described [24].

The study was approved by the local ethics committees (IRB-No. 4208, 21.04.2010, amendment approved 06.02.2017, Medical Faculty, Friedrich-Alexander University Erlangen-Nürnberg (FAU), Germany, and IRB-No. 2017-079-f-S, 05.07.2017, Medical Council Westfalen-Lippe and Westfälische Wilhelms-Universität Münster, Germany). All participants signed the written informed consent according to the Declaration of Helsinki.

### Statistical analysis

Normality of data was tested by Shapiro–Wilk test and variance homogeneity by Levene test. Mann–Whitney *U* test was used to verify group differences regarding anthropometric variables (age, weight, and height). Gender differences between groups were evaluated using Chi-squared test. Since gait parameters were not distributed normally, Mann–Whitney *U* test and Kruskal–Wallis test (for group comparisons with TFC) were used to identify group differences in gait characteristics. Kruskal–Wallis test was followed by Dunn-Bonferroni post-hoc tests in order to analyze separate differences between the groups. Besides *p* values (significance level *α* = 0.05), Cohens *d* is presented as measure of effect size. Correlation analysis between gait parameters and clinical scores (UHDRS TMS and TFC) was performed using Spearman’s rank correlation (*r*_Sp_). Moreover, gait characteristics were compared in TFC groups based on a previously described classification [25]. TFC scores 11–13 were grouped in stage I (early), 7–10 stage II (moderate), and 0–6 stage III (advanced). Due to small sample sizes in severely affected HD patients (TFC scores 1–2 and 0), patients with TFC scores from 0 to 6 were combined in one category (III).

## Results

### Between-group differences in gait parameters

**Fig. 1:**
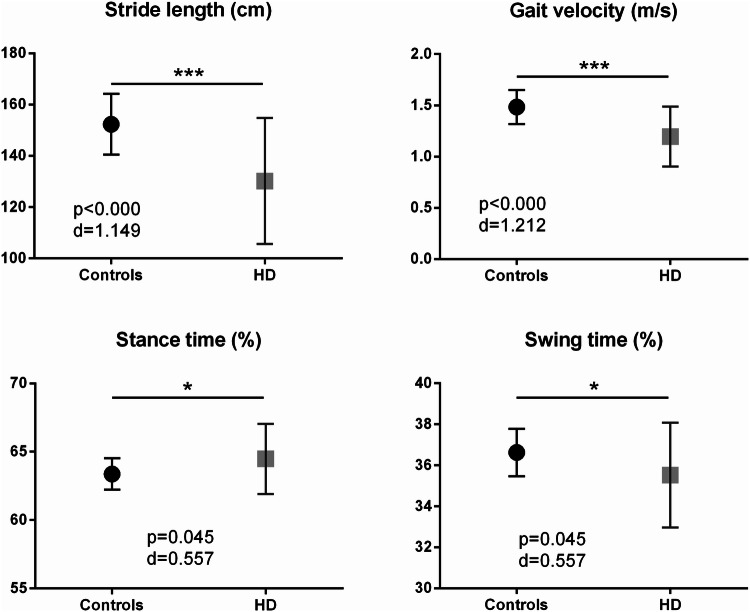
Spatio-temporal gait parameters: stride length (cm), gait velocity (m/s), stance time (%), and swing time (%) show significant differences between patients with HD and controls (*p <0.05; ***p < 0.001)

Specific gait parameters such as stride length (mean ± SD; HD: 1.30 ± 0.25 m, controls: 1.52 ± 0.12 m, delta *Δ* = − 15%, *p* < 0.000) and gait velocity (HD: 1.20 ± 0.29 m/s, controls: 1.48 ± 0.16 m/s, *Δ* = − 19%, *p* < 0.000) were severely reduced, as expected. Stride time (HD: 1.11 ± 0.15 s, controls: 1.03 ± 0.08 s, *Δ* =  + 7%, *p* = 0.008) and stance time (HD: 64.5 ± 2.55%, controls: 63.4 ± 1.16%, *Δ* =  + 2%, *p* = 0.045) were significantly increased in patients with HD compared to controls (Fig. 1).

**Fig. 2:**
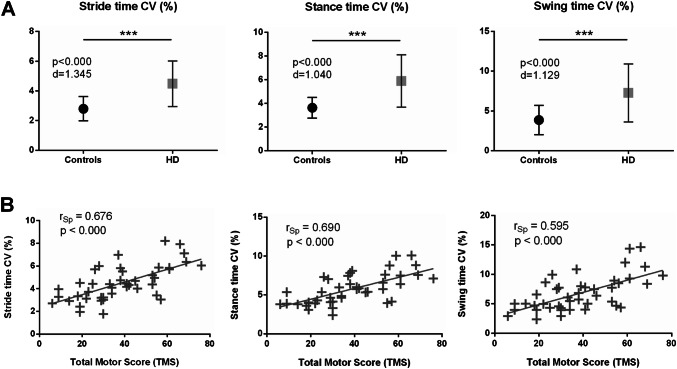
**a** Variability in stride time, stance time and swing time is significantly increased in patients with HD compared to healthy controls (***p < 0.001). *d* Cohen’s *d* effect size, *CV* coefficient of variance. **b** Stride time CV, stance time CV, and swing time CV of patients with HD correlate to UHDRS total motor score, *r*_*Sp*_ Spearman’s rank correlation coefficient

Group differences for parameters representing irregularity of gait were more pronounced in HD in comparison to controls: stride time CV (HD: 4.46 ± 1.55%, controls: 2.80 ± 0.81%, *Δ* =  + 37%, *p* < 0.000), swing time CV (HD: 5.59 ± 2.51%, controls: 3.28 ± 1.43%, *Δ* =  + 41%, *p* < 0.000), stance time CV (HD: 3.06 ± 1.26%, controls: 1.91 ± 0.91%, *Δ* =  + 38%, *p* < 0.000), stride length CV (HD: 7.96 ± 2.13%, controls: 6.59 ± 3.08%, *Δ* =  + 17%, *p* = 0.001), and gait velocity CV (HD: 8.79 ± 2.43%, controls: 7.39 ± 2.84%, *Δ* =  + 16%, *p* = 0.001) (Fig. 2). Cohen’s *d* effect sizes showed the largest differences between groups for the gait variability parameter stride time CV (Cohen’s *d* = 1.345), followed by swing time CV (*d* = 1.129), and stance time CV (*d* = 1.040). The effect size for stride length (*d* = 1.149) and gait velocity (*d* = 1.212) was strong but did not reach those of stride time CV (Supplementary Table T1).

### Correlation analysis between gait parameters and clinical scores

Parameters representing gait variability showed moderate to strong correlations to UHDRS TMS: stride time CV (*r*_Sp_ = 0.676, *p* ≤ 0.000), stance time CV (*r*_Sp_ = 0.690, *p* ≤ 0.000), swing time CV (*r*_Sp_ = 0.595, *p* ≤ 0.000), stride length CV (*r*_Sp_ = 0.416, *p* = 0.006), and gait velocity CV (*r*_Sp_ = 0.579, *p* ≤ 0.000). Stride length and gait velocity showed moderate inverse correlations to UHDRS TMS: stride length (*r*_Sp_ = − 0.549, *p* ≤ 0.000), and gait velocity (*r*_Sp_ = − 0.478, *p* = 0.001).

**Fig. 3:**
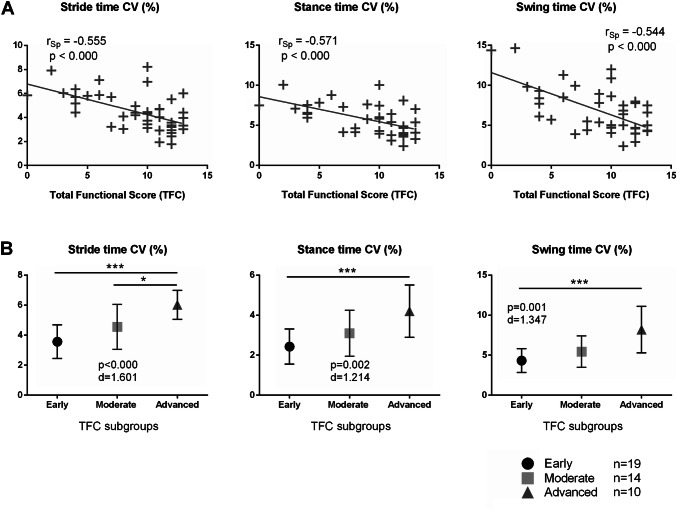
**a** Stride time CV, stance time CV and swing time CV of patients with HD correlate to TFC. *CV* coefficient of variance, *r*_*Sp*_ Spearman’s rank correlation coefficient. **b** Stride time CV, stance time CV and swing time CV of patients with HD grouped by TFC score (*p < 0.05; ***p < 0.001). Early HD = TFC score 11–13, moderate HD = TFC 7–10, advanced HD = TFC 0–6. *d* Cohen’s *d* effect size

Furthermore, the objective gait variability measures reflected the patients’ functional abilities according to TFC by moderate inverse correlations: stride time CV (*r*_Sp_ = − 0.555, *p* ≤ 0.000), stance time CV (*r*_Sp_ = − 0.521, *p* ≤ 0.000), swing time CV (*r*_Sp_ = − 0.561, *p* ≤ 0.000), stride length CV (*r*_Sp_ = − 0.468, *p* = 0.002), and gait velocity CV (*r*_Sp_ = − 0.628, *p* ≤ 0.000). Graphs to this correlation analysis are presented in Figs. 2 and 3. Group comparisons between TFC subgroups (early, moderate, advanced) revealed highly significant differences in stride time CV (*p* < 0.001, *d* = 1.601), stance time CV (*p* = 0.002, *d* = 1.214), swing time CV (*p* = 0.001, *d* = 1.347), stride length CV (*p* = 0.010, *d* = 0.935), and gait velocity CV (*p* < 0.000, *d* = 1.613). Dunn-Bonferroni post-hoc tests showed differences between the TFC subgroups for stride time CV (early vs. advanced: *p* < 0.000, moderate vs. advanced: *p* = 0.045), stance time CV (early vs. advanced: *p* = 0.001), swing time CV (early vs. advanced: *p* < 0.000), stride length CV (early vs. advanced: *p* = 0.008), and gait velocity CV (early vs. advanced: *p* < 0.000) (Fig. 3b). Importantly, stride time CV was the sole parameter detecting differences between moderate and advanced HD patients.

## Discussion

The aim of this study was to objectively assess characteristic features of gait in HD patients compared to age- and gender-matched controls using mobile sensor-based gait analysis. In particular, gait parameters were correlated to clinical scores (TMS and TFC) in order to clinically validate these objective parameters. The main finding demonstrated that gait variability parameters representing the disease-characteristic irregularity of gait were the most relevant parameters correlating with TMS and TFC. Increased stride time CV in HD patients showed the largest effect size in comparison to gait of controls. Moreover, we observed severely reduced stride length and gait velocity as well as significantly increased stride time, and stance time in HD patients compared to healthy controls, albeit, with smaller effect sizes.

### Gait impairment in HD

Objective gait measures of the present study showed that HD patients walk slower (− 19%) and with shorter steps (− 15%) compared to matched healthy controls. Importantly, we observed that gait variability representing the HD-characteristic irregular gait signature is substantially increased in HD patients (17–41%). Herewith, we confirm previous findings, however with mobile, sensor-based technology in comparison to stationary systems [12]. Gait variability is serving as a quantitative marker for quality of gait and falls [8, 11, 26]. With regard to stride length and gait velocity, we confirm results from previous small-cohort studies reporting motor impairment reflected by gait parameters, however with more statistical power due to larger sample sizes in our dual-center study with age- and gender-matched cohorts. Intriguingly, we confirm by applying mobile sensor-based technology that especially gait variability parameters characterize at best HD-typical gait. The largest effect size for differences between gait in HD patients and matched healthy controls was noticed for stride time CV (*d* = 1.345) followed by gait velocity (*d* = 1.212), stride length (*d* = 1.149), swing time CV (*d* = 1.129), and stance time CV (*d* = 1.040). These results suggest that gait parameters derived from sensor-based gait analysis serve as an objective, digital biomarker for gait patterns in HD. In particular, increased gait variability appeared to be characteristic for HD patients. This finding is in line with previous studies showing increased movement variability of the upper extremity by investigating finger tapping, and grasp force in HD [27–30]. Grip force variability is discussed as an objective measure to evaluate motor deficits and reflect disease progression in HD [31]. Our results identify gait variability as a disease-characteristic signature and an important digital biomarker in terms of gait dysfunction in HD patients, similar to grip force variability as an objective and quantitative outcome for motor deficits in the upper extremity. In a small longitudinal study with ten pre-manifest gene carriers, gait variability has increased within the first year from baseline and may be a likely marker for disease progression [32]. Future studies should further investigate sensor-based gait variability parameters longitudinally in order to determine whether this measure may be an appropriate progression marker for HD. In addition, gait variability should be investigated in premanifest HD patients in order to evaluate this measure as potential early-detection marker for clinical symptoms. Previous work supports that it is worth investigating quantitative gait in premanifest HD patients [8].

### Sensor-based gait variability parameters reflect disease severity

Gait variability parameters derived from sensor-based gait analysis strongly correlated to the established clinical motor ratings TMS and TFC. This finding indicates that CV measures are an objective mirror of the ordinal clinical motor examinations. Instrumented quantitative measures are discussed as digital biomarkers and complementary outcomes since clinical rating scales are limited due to inter- and intra-rater variability as well as rater-induced placebo effects in clinical trials [33–35]. A meta-analysis based on 800 patients with Parkinson’s disease and 854 healthy subjects provided evidence that a stride time variability larger than 2.4% discriminates healthy from pathological gait [36]. Sensor-based gait variability parameters were identified as very important objective measure in differentiating patients with atypical parkinsonian disorders (larger gait variability) from patients with sporadic Parkinson’s disease [22]. In HD, it has been shown that the instrumented measure of variability in grasp forces strongly correlated with motor performance assessed by TFC (*r* = − 0.712) and TMS (*r* = 0.841) suggesting that movement variability is a key feature of motor impairment in HD [29]. In a similar way, quantitative assessments of chorea in HD patients has shown to be feasible, easy applicable and may improve sensitivity and reliability of motor end points in clinical studies [34]. Therefore, instrumented quantitative data provide important measurements complementing established clinical ratings and may be useful for the evaluation of motor deficits in HD.

### Wearable sensor system as easy-to-apply tool for objective gait assessment

We observed that wearable sensors provide metric, granular information in regard to gait impairment in HD patients. Easy-to-apply wearable systems are able to provide stride-by-stride variance parameters which have in this study been demonstrated to play an important role in HD gait. Sensor-based gait data correlated to established clinical scores and, therefore, indicates that inertial sensors are able to reflect the rating of clinical experts. This is in line with previous studies in other basal ganglia diseases like Parkinson’s disease [13–15] and atypical Parkinsonian disorders [16] reporting that wearable sensor systems support the clinical workup by objective, quantitative data. In HD, previous studies observed that inertial sensors are able to differentiate between HD patients and healthy controls [20]. They have been validated for the analysis of gait characteristics in HD patients by comparing spatio-temporal gait parameters derived from sensors with those from instrumented gait mats [6, 37]. In contrast to mats, future mobile sensor technology may be used in home-monitoring scenarios [17, 18] over several hours in order to provide a comprehensive and long-lasting monitoring tool complementing established short-lasting clinical examinations in the outpatient units. In a pilot study, the feasibility was shown to use wearable sensors in the hospital and in the home-environment of HD patients [38]. Future studies need to further investigate the application of inertial sensors as objective measure to detect  motor impairment  more precisely and outside the lab. In particular, it is interesting to record irregular movements in the upper and lower extremity of HD patients in everyday life.

## Conclusion

In conclusion, our data demonstrate that sensor-based gait variability parameters were identified as the clinically most relevant digital biomarker for gait impairment in HD. They showed the largest effect size in group comparison and strongly correlated to established clinical scores (TFC, TMS). Thus, sensor-based gait variability represents the irregularity of gait characteristic for HD and reflects disease severity. Moreover, we observed reduced stride length and gait velocity as well as increased stride time and stance time in HD patients compared to age- and gender-matched controls. Our cohort-based clinical validation study confirmed the clinical relevance of a detailed and objective gait analysis in HD. As the altered sensor-based gait variability parameters of this study may mirror disease progression according to TFC, there is a strong need for longitudinal studies validating these initial findings. The transfer from cross-sectional studies to disease monitoring including long-term recordings at patients’ home and individual care should be the focus of future studies using sensor-based gait analysis.

## Electronic supplementary material

Below is the link to the electronic supplementary material.

Supplementary file1 (PDF 256 kb)
