# Correction to: Gait variability as digital biomarker of disease severity in Huntington’s disease

**DOI:** 10.1007/s00415-020-09789-1

**Published:** 2020-03-27

**Authors:** Heiko Gaßner, Dennis Jensen, Franz Marxreiter, Anja Kletsch, Stefan Bohlen, Robin Schubert, Lisa M. Muratori, Bjoern Eskofier, Jochen Klucken, Jürgen Winkler, Ralf Reilmann, Zacharias Kohl

**Affiliations:** 1 Department of Molecular Neurology, University Hospital Erlangen, Friedrich-Alexander University Erlangen-Nürnberg (FAU), Schwabachanlage 6, 91054 Erlangen, Germany; 2 George-Huntington Institute (GHI) GmbH, Münster, Germany; 3 Rehabilitation Research and Movement Performance Laboratory (RRAMP Lab), Stony Brook University, Stony Brook, NY USA; 4 Machine Learning and Data Analytics Lab, Friedrich-Alexander University Erlangen-Nürnberg (FAU), Erlangen, Germany; 5 Medical Valley-Digital Health Application Center GmbH, Bamberg, Germany; 6 Fraunhofer Institute for Integrated Circuits IIS, Erlangen, Germany; 7 Department of Radiology, University of Muenster, Muenster, Germany; 8 Department of Neurodegenerative Diseases and Hertie-Institute for Clinical Brain Research, University of Tuebingen, Tuebingen, Germany; 9 Center for Rare Diseases Erlangen, University Hospital Erlangen, Erlangen, Germany; 10 Department of Neurology, University of Regensburg, Regensburg, Germany

## Correction to: Journal of Neurology 10.1007/s00415-020-09725-3

The original version of this article unfortunately contained a mistake. The name of one author is not presented correctly in the author group.

The correct name should be Franz Marxreiter.

