# Supplementary material for: Gait variability as digital biomarker of disease severity in Huntington’s disease
Source: J Neurol. 2020 Feb 11;267(6):1594–601. doi: 10.1007/s00415-020-09725-3 (PMC7293689; doi:10.1007/s00415-020-09725-3)
Supplement: Supplementary file 1 — Supplementary file1 (PDF 256 kb) [file 415_2020_9725_MOESM1_ESM.pdf]

Supplementary Figure

Manuscript title: Objective sensor-based gait analysis: Gait variability as digital biomarker of disease severity in Huntington's disease

Journal of Neurology

Authors: Gaßner H, Jensen D, Marxreiter F, Kletsch A, Bohlen S, Schubert R, Muratori LM, Eskofier B, Klucken J, Winkler J,Reilmann R, Kohl Z

Corresponding author: Zacharias Kohl, MD, present email-address: Zacharias.Kohl@klinik.uni-regensburg.de

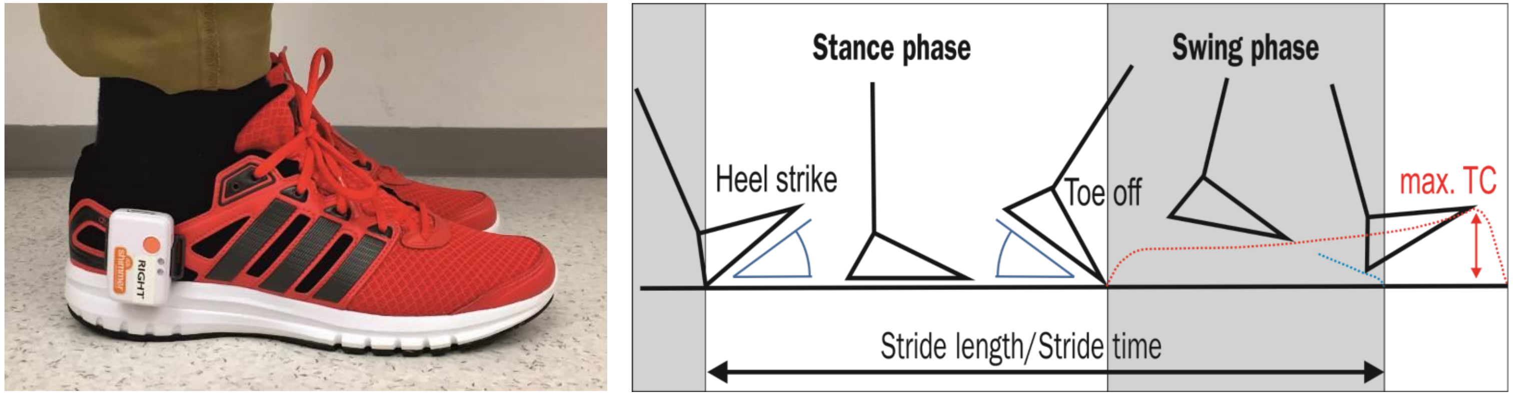

A

B

Figure S1 A) Inertial Sensors laterally attached to the heel of running shoes; lateral view and B) Overview of calculated gait parameters (Raccagni, Gaßner et al. 2018; Suppl. material); gait velocity was calculated in addition. Max. TC = maximum toe clearance

Supplementary Table T1

Manuscript title: Objective sensor-based gait analysis: Gait variability as digital biomarker of disease severity in Huntington's disease

Journal of Neurology

Authors: Gaßner H, Jensen D, Marxreiter F, Kletsch A, Bohlen S, Schubert R, Muratori LM, Eskofier B, Klucken J, Winkler J,Reilmann R, Kohl Z

Corresponding author: Zacharias Kohl, MD, present email-address: Zacharias.Kohl@klinik.uni-regensburg.de

Gait parameters	HD patients		Controls		P	Cohens d
	Mean	SD	Mean	SD		
Stride time (s)	1.11	0.15	1.03	0.08	0.008	0.674
Swing time (%)	35.5	2.55	36.6	1.16	0.045	0.557
Stance time (%)	64.5	2.55	63.4	1.16	0.045	0.557
Stride length (m)	1.30	0.25	1.52	0.12	0.000	1.149
Gait velocity (m/s)	1.20	0.29	1.48	0.16	0.000	1.212
Stride time CV (%)	4.46	1.55	2.80	0.81	0.000	1.345
Swing time CV (%)	5.59	2.51	3.28	1.43	0.000	1.129
Stance time CV (%)	3.06	1.26	1.91	0.91	0.000	1.040
Stride length CV (%)	7.96	2.13	6.59	3.08	0.001	0.516
Gait velocity CV (%)	8.79	2.43	7.39	2.84	0.001	0.531

CV=Coefficient of Variance
